# Keenness of the Senses

**Published:** 1869-11

**Authors:** 


Keeness of the Senses.—Saunderson the mathematician, lost his sight in 1683—when only one year old—after a severe attack of the small-pox. But spite of his complete blindness, he gave himself up to the assiduous duty of the sciences, and finally lectured at the University of Cambridge, on mathematics and optics, with wonderful success. His sense of touch was exquisitely fine. Thus in a collection of Roman medals, he could distinguish the genuine from the false, although the latter were often so admirably counterfeited as to deceive those who examined them with their eyes. By the different feeling of the air on his face, he could tell when an object was placed before him. And his hearing was so accurate in seizing and appreciating the slightest sounds, that he could determine the height of any chamber into which he was introduced, and his distance from the wall.

